# Chromosome 16p13.3 Contiguous Gene Deletion Syndrome including the *SLX4*, *DNASE1*, *TRAP1*, and *CREBBP* Genes Presenting as a Relatively Mild Rubinstein–Taybi Syndrome Phenotype: A Case Report of a Saudi Boy

**DOI:** 10.1155/2020/6143050

**Published:** 2020-01-09

**Authors:** Mohammad M. Al-Qattan, Zuhair A. Rahbeeni, Zuhair N. Al-Hassnan, Abdulaziz Jarman, Atif Rafique, Nehal Mahabbat, Faris A. S. Alsufayan

**Affiliations:** ^1^Division of Plastic Surgery, King Saud University, Riyadh, Saudi Arabia; ^2^Division of Plastic Surgery, King Faisal Specialist Hospital and Research Center, Riyadh, Saudi Arabia; ^3^Department of Medical Genetics, King Faisal Specialist Hospital and Research Center, Riyadh, Saudi Arabia; ^4^Prince Mohammed Bin Abdulaziz Hospital, Riyadh, Saudi Arabia

## Abstract

The classic Rubinstein–Taybi syndrome Type 1 (RSTS1, OMIM 180849) is caused by heterozygous mutations or deletions of the *CREBBP* gene. Herein, we describe the case of a Saudi boy with chromosome 16p13.3 contiguous gene deletion syndrome (OMIM 610543) including the *SLX4*, *DNASE1*, *TRAP1*, and *CREBBP* genes, but presenting with a relatively mild RSTS1 syndrome phenotype. Compared with previously reported cases with severe phenotypes associated with 16p13.3 contiguous gene deletions, our patient had partial deletion of the *CREBBP* gene (with a preserved 5′ region), which might explain his relatively mild phenotype.

## 1. Introduction

Rubinstein–Taybi syndrome Type 1 (RSTS1, OMIM 180849) is a rare autosomal dominant syndrome, and most patients with this syndrome (over 99% of cases) present *de novo* heterozygous mutations or deletions of the *CREBBP* gene [1]. There are three distinct phenotypes of RSTS1, namely, the classic, mild, and very severe phenotypes. The classic phenotype is characterized by three main features, namely, intellectual disability, broad angulated thumbs and halluces, and characteristic facial dysmorphism such as highly arched eyebrows, a broad nose, a columella hanging below the alae nasi, and a pouting lower lip. Other features include cardiac defects, renal abnormalities, cleft palate, bifidity of the tongue tip, midline grooving of the lower lip, and increased risk of developing tumors [2]. The classic phenotype is usually caused by deletions or truncating mutations of the *CREBBP* gene. In contrast, the mild phenotype (also known as incomplete RSTS1) is usually caused by missense *CREBBP* mutations and is characterized by the presence of mild facial features and broad thumbs and halluces. There is no intellectual disability, and other systemic features are usually absent [3, 4]. Finally, a very severe and often fatal phenotype of RSTS1 syndrome is known as the chromosome 16p13.3 contiguous deletion syndrome [5] (OMIM 610543). Besides the classic features, patients with this syndrome exhibit severe life-threatening infections, severe mental retardation, severe neonatal convulsions, multifocal hypsarrhythmia, hypoplastic left heart, polysplenia, and laterality disturbance. This very severe phenotype is caused by large deletions including the *CREBBP* gene and the 3′ adjacent genes, *viz.*, *DNASE1* and *TRAP1* [5]. It has been reported that patients with large deletions including the *CREBBP* gene but without these two 3′ adjacent genes exhibit a classic and not a severe phenotype [5]. Herein, we report a case of Saudi boy with chromosome 16p13.3 contiguous gene deletion syndrome including the *SLX4*, *DNASE1*, *TRAP1*, and *CREBBP* genes, but presenting with a relatively mild phenotype of Rubinstein–Taybi syndrome.

## 2. Case Report

**Figure 1:**
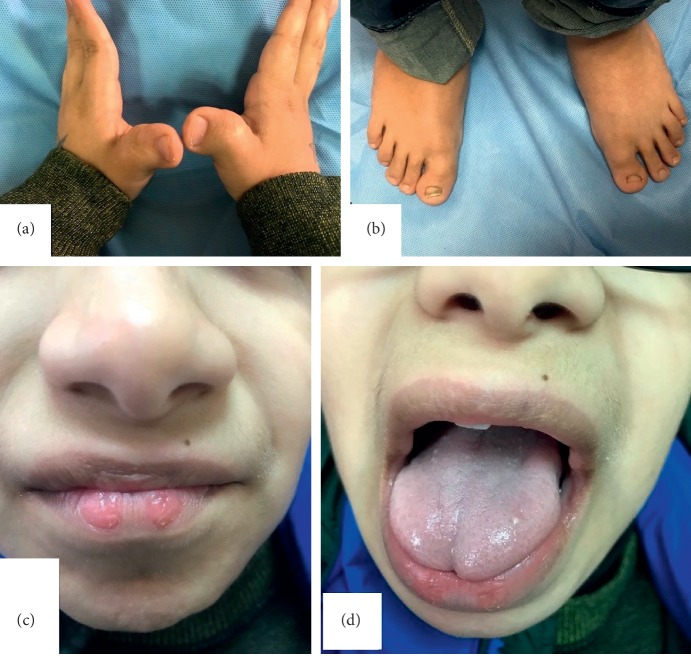
(a) Broad and radially angulated thumbs. (b) Broad halluces. (c) Bulbous nasal tip, a columella hanging below the alae nasi, and a pouting lower lip. The two paramedian rounded areas in the lower lip are vermilion elevations with no fistulae. (d) Bifid tip of the tongue.

A 7-year-old Saudi boy presented to the Hand Clinic with bilateral thumb deformities. The thumbs were broad and radially angulated (Figure 1(a)). The feet also showed broad halluces (Figure 1(b)). There was mild intellectual disability. Mild facial dysmorphism was also observed, including mild arching of the eyebrows, a broad nose with a bulbous nasal tip, a columella hanging below the alae nasi, pouting lower lip with two paramedian vermilion elevations in the lip, and a bifid tip of the tongue (Figures 1(c) and 1(d)). His history included mild right hydronephrosis, left nasolacrimal obstruction successfully treated by probing and stenting, and a cardiac surgery for the repair of total anomalous pulmonary venous drainage (TAPVD). The clinical diagnosis was a classic RSTS1 syndrome phenotype.

After obtaining informed consent, genomic DNA was extracted from the peripheral blood of both parents and the patient, who was their only child. Genetic testing was initiated with a SNP microarray which revealed a 398.43 kb pathogenic deletion in the chromosome region 16p13.3 from positions 3,358,012–3,796,442 which encompasses three OMIM Morbid Map genes including *SLX4*, *DNASE1*, and *CREBBP*. The genomic microarray platform used was the CytoScan HD SNP array (Affymetrix) with the genome build: NCBI 37/hg19 (2009). This platform contains approximately 1.9 million copy number probes and 743,000 SNP probes. The assay will detect genomic gains/losses of approximately 50 kb across the genome and 20 kb in clinically significant targeted genes. Further confirmation by copy number analysis using MLPA as well as sequencing was proceeded with and confirmed the patient to be heterozygous for a partial deletion of the CREBBP gene. This was found to be involving exons 22 to 31, thus sparing the 5′ end of the CREBBP gene. Neither parent had the deletion. At the resolution allowed by the SNP array platform used, the deletion seems to be homogeneous (not mosaic).

## 3. Discussion

The most significant finding in our patient is the presence of a large deletion including the *CREBBP* gene and the adjacent 3′ genes, yet he presented a relatively mild RSTS1 phenotype.  Table 1 shows the genomic coordinates of the deleted genes in our patient. Homozygous mutations of the *SLX4* gene cause Fanconi anemia, complementation group P (OMIM: 613951) [6, 7]. This is not surprising as SLX4 is a coordinator of structure-specific endonucleases and is involved in DNA repair [7,8]. Our patient had no Fanconi anemia because the *SLX4* gene deletion was heterozygous. The second gene deleted in our patient was the *DNASE1* gene, which encodes an endonuclease known as deoxyribonuclease 1 [9]. Heterozygous mutations in *DNASE1* cause systemic lupus erythematous in humans and animal models [10, 11]. Systemic lupus erythematous is an autoimmune disorder, which is associated with an increased risk of infections. Furthermore, Bartsch et al. [5] proposed that the reduced clearance of nuclear protein complexes (as a result of DNASE1 deficiency) might explain the severe neonatal convulsions associated with the chromosome 16p13.3 contiguous gene deletion syndrome. Our patient had no history of severe infections or epilepsy despite the deletion of *DNASE1*. The third gene deleted in our patient was the *TRAP1* gene, which encodes the tumor necrosis factor receptor-associated protein 1. The TRAP1 protein is a mitochondrial ATP-binding protein and is involved in maintaining mitochondrial function [12]. TRAP1 is also a heat shock protein 90-related protein, and hence, it acts as a molecular chaperone. Furthermore, TRAP1 interacts with the retinoblastoma protein [13]. However, the *TRAP1* gene has not been associated with human diseases.

The main question that arises is regarding the relatively mild phenotype in our patient despite the deletion of the *CREBBP* gene and the 3′ adjacent genes. One possible explanation might be related to the preservation of the 5′ region of the *CREBBP* gene in our patient (Table 1). All patients reported by Bartsch et al. [5] with a severe phenotype had complete deletion of the *CREBBP* gene. Isolated deletion of the 5′ region of the *CREBBP* gene is known to result in the classic RSTS1 phenotype (Case #5, Bartsch et al. [5]). Hence, the preservation of the 5′ region of the *CREBBP* gene might have modified the phenotype despite the complete deletion of the adjacent 3′ genes (Table 1). To support our hypothesis, we reviewed the Decipher database (https://decipher.sanger.ac.uk) for partial pathogenic deletions of the *CREBBP* gene. We identified two patients with chromosome 16p13.3 contiguous gene deletion syndrome including the *SLX4*, *DNASE1*, *TRAP1*, and *CREBBP* genes, but with preserved 5′ region of the *CREBBP* gene (which is similar to our case). The first patient (patient ID: 283606) had the deletion 16: 2,828,888–3,817,850, and the second patient (patient ID: 355716) had the deletion 16: 3,363,349–3,879,467. Both patients would seem to have a relatively mild RSTS1 phenotype, without any of the distinct severe features of the chromosome 16p13.3 contiguous gene deletion syndrome.

The OMIM catalogue distinguishes the Rubinstein–Taybi Type 1 syndrome (180849) from the 16p13.3 deletion syndrome (610543). The first is caused by mutations in the *CREBBP* gene, while the second is considered a contiguous gene syndrome due to a deletion of a trait of the short arm of chromosome 16, containing the *CREBBP* (600140), *DNASE1* (125505), and *TRAP1* (*HSP75*; 606219) genes. However, the most recent literature has questioned this distinction. Although Bartsch et al. [5] suggested that patients with severe RSTS and large deletions had a distinct contiguous gene syndrome, several other authors did not confirm these findings. Stef et al. [14] did not find a correlation between the size of the deletion and the patients' phenotypic severity. Gervasini et al. [15] reported on several patients with 16p13.3 contiguous gene deletion syndrome without severe symptoms or fatal infections despite the complete deletion of the *DNASE1* gene. According to Rusconi et al. [16], genotype/phenotype correlations indicated that patients with larger deletions did not always have a more severe phenotype than those with smaller deletions, suggesting that the idea of a contiguous gene deletion syndrome in such patients, as proposed by Bartsch et al. [5], may not be accurate.

The OMIM catalogue also describes a third category known as the 16p13.3 contiguous gene duplication syndrome (OMIM 613458). In addition to the duplication of the *CREBBP* gene, several other adjacent genes (such as the *DNASE1*, *SLX4*, *MEFV*, and *THOC6* genes) are also duplicated. These patients have all the classic facial features of Rubinstein–Taybi Type 1 syndrome. Most patients also have mild to moderate intellectual disability. However, the thumbs are proximally displaced instead of being broad and angulated [17, 18].

Finally, patients with isolated mosaic deletions in the *CREBBP* gene are known to have a mild Rubinstein–Taybi phenotype [15].  Table 2 summarizes all the phenotypes related to the *CREBBP* gene.

In conclusion, we describe a case of chromosome 16p13.3 contiguous gene deletion syndrome including the *SLX4*, *DNASE1*, *TRAP1*, and *CREBBP* genes, but presenting with a relatively mild RSTS1 syndrome phenotype. Compared with previously reported cases of severe phenotypes, our patient had partial deletion of the *CREBBP* gene (preserving the 5′ region of the gene), which might explain his relatively mild phenotype.

## Figures and Tables

**Table 1:** The genomic coordinates of the deleted genes of interest in our patient.

Gene	Genomic coordinates	Status in our patient with the deletion from positions 3,358,012 to 3,796,442
*SLX4*	Chr16: 3,631,184–3,661,585	Completely deleted
*DNASE1*	Chr16: 3,702,940–3,708,096	Completely deleted
*TRAP1*	Chr16: 3,708,038–3,767,598	Completely deleted
*CREBBP*	Chr16: 3,775,056–3,930,121	Partially deleted (the 5′ region of the *CREBBP* gene is not deleted)

**Table 2:** Phenotypes related to the *CREBBP* gene.

Given name	OMIM	Deletion/mutation/duplication of the CREBBP gene	Phenotype
Classic Rubinstein–Taybi Type I syndrome	180849	Usually deletions or truncating mutations	Characteristic facial dysmorphism, variable intellectual disability, broad/angulated thumbs, and halluces. Other systemic abnormalities (such as cardiac defects) are frequent.
The incomplete (mild) Rubinstein–Taybi Type I syndrome	—	Usually missense or mosaic mutations	The facial and the thumb/big toe features are similar to the classic type. However, there is usually mild or no intellectual disability. Other systemic defects are usually absent.
16p13.3 contiguous deletion syndrome	610543	Large deletions including the *CREBBP* gene and the 3′ adjacent genes	Controversial issue in the literature. Some believe that the phenotype is more severe and is associated with severe/fatal infections, while others could not find a correlation between the size of the deletion and the phenotypic severity. Our case was unique because of the relatively mild phenotype.
16p13.3 contiguous duplication syndrome	613458	Duplication of the *CREBBP* and other adjacent genes	Classic facial dysmorphism and mild to moderate intellectual disability. However, the thumbs are proximally displaced instead of being broad and angulated.
